# Preparation of Powdered Infant Formula: Could Product's Safety Be Improved?

**DOI:** 10.1097/MPG.0000000000002100

**Published:** 2018-07-30

**Authors:** Marina Nadia Losio, Enrico Pavoni, Guido Finazzi, Carlo Agostoni, Paolo Daminelli, Elena Dalzini, Giorgio Varisco, Stefano Cinotti

**Affiliations:** ∗ Istituto Zooprofilattico Sperimentale della Lombardia e dell’Emilia Romagna “B. Ubertini,” Brescia; † Pediatric Intermediate Care Unit, Fondazione IRCCS “Ca’ Granda” Ospedale Maggiore Policlinico, DISCCO, University of Milan, Milan, Italy.

**Keywords:** *Cronobacter sakazakii*, outbreaks., powdered formula, *Salmonella* spp

## Abstract

The recent outbreak of *Salmonella* Agona linked to the consumption of infant formula (powdered formula) has rekindled the attention about the correct procedures for preparation and use of these products. International guidelines have already been published so far, particularly in association with *Cronobacter sakazakii* in early 2000s. FAO/WHO suggested to reconstitute formula with water at no less than 70°C. We therefore contaminated powdered formula with low levels of *Salmonella* spp and *C sakazakii* to evaluate the pathogens inactivation during the formula preparation using water at 70°C. In these conditions we observed a survival of both pathogens, indicating that the suggested recommendations may be not enough to guarantee the safety of this product. Higher temperatures are needed to reduce the biological risk, even if it may be not easily realized in actual domestic conditions. Moreover, the impact on the nutritional value of reconstituted formulas should be evaluated.

**What Is Known**

- Powdered formula has been associated with serious illness due to infections with *Salmonella* spp and *Cronobacter sakazakii.*
- International guidelines have been published to indicate how to prepare powdered formula, using water not below 70°C.

**What Is New**

- In practical use, starting from water at 70°C, maximum temperatures registered in 200 mL of reconstituted formula were between 57.5 and 60°C.
- These conditions may not ensure inactivation of pathogens.
- Water at higher temperature must be considered to prepare powdered formula, to improve the food safety, but pros and cons need further evaluation.

*Salmonella* spp. and *Cronobacter sakazakii* are ubiquitous Gram-negative, facultative anaerobic, motile, non-spore–forming bacteria that cause human disease (1). Since August 2017, an outbreak of *Salmonella* Agona linked to the consumption of infant formula (powdered formula) has been ongoing in France. As of January 11, 2018, the outbreak affected 39 infants (children <1 year of age): 37 in France, 1 in Spain and 1 in Greece, but new cases may be detected. Epidemiological investigations in humans and traceability investigations in food, identified seven different brands of infant formula from a single processing company in France as the vehicles of infection (2). No other common foods or drinks were identified among cases. A variety of types of water (tap and different brands of bottled water) were used to prepare these products, and no evidence was given that infections were related to inappropriate methods for preparing and handling powdered infant formula by the caregivers (3). Rather, some weaknesses could have been happened in the production process (2). Salmonella infections due to the consumption of contaminated infant formula can occur in healthy term infants, while *C sakazakii* mainly occur in preterm, unhealthy, immunocompromised infants, hospitalized in neonatal intensive care units (4,5).

The current consolidated Reg. CE 2073/2005 (6) states for *Salmonella* spp and for *Cronobacter* spp (*Enterobacter sakazakii*) the complete absence in 30 samples of 25 and 10 g, respectively, during the shelf-life in infant formula to be distributed.

Starting early 2000s, following an outbreak of *C sakazakii*(7) increasing attention has been addressed to the safety issue of reconstituted infant formulas, particularly focusing on products and recipients (8). In 2007, World Health Organization (WHO) in collaboration with the Food and Agriculture Organization of the United Nations (FAO), published the guidelines about the safe preparation, storage and handling of powdered infant formula. The recommendations in this guidance document were largely based on the quantitative risk assessment for *C sakazakii* and not evidence based, establishing that the inclusion of a pathogenic lethal step at preparation (eg, reconstitution of formula with water ≥70°C), and a decrease during holding and feeding time would effectively reduce the risk. No risk assessment was carried out for *Salmonella* spp, but the expert group reported that the basic risk control principles for *C sakazakii* would also hold for *S enterica*(9).

In our study we monitored the time/temperature profile of formula and the survival of *Salmonella* spp and *C sakazakii* during the preparation of powdered infant formula following the procedure suggested by FAO/WHO (10) and an alternative procedure.

## METHODS

### Formula Contamination

Three different *Salmonella* spp strains (*Salmonella* Typhimurium ATCC 14028, *Salmonella* Abortus ovis CIP 55132 from *Institut Pasteur*, *Paris* and *Salmonella* Agbeni, wild strain) and one *C sakazakii* strain (CIP 103183 from Institut Pasteur, Paris, France), were used to contaminate powdered formula (commercial formula). Before use, the individual *Salmonella* strains were serially diluted and combined in equal volumes to obtain a multistrain cocktail. *C sakazakii* strain was separately diluted. Sterile plastic 250 mL bottles containing 30 g of powdered formula were separately contaminated with 2 mL of each pathogen suspension (to reach a final inoculum level of about 1 to 1.5 log CFU/mL in reconstituted formula). Another bottle containing 30 g of powdered formula was prepared to check the time/temperature profile during the test.

### Preparation Procedures

Formula was reconstituted with 200 mL (15% w/v) of hot water from commercial plastic bottles. To simulate the procedure suggested by FAO/WHO, water was previously brought to a boil (500 mL) and allowed to cool at room temperature up to 70°C; to evaluate an alternative procedure, water was previously brought to a boil (500 mL) and allowed to cool at room temperature for 10 minutes. In both cases, at the end of the reconstitution phase the product was mixed by stirring the bottle for 120 seconds and then it was stored at room temperature for 2 hours (maximum storage time suggested for reconstituted product). A positive control was prepared adding water at room temperature (∼24°C) to contaminated formula, to calculate the initial inoculum level for each pathogen. Three independent replicates were performed for each preparation procedure.

### Sampling Times and Analyses

Both pathogens were enumerated in positive control by plate count methods in Blood Agar Base (incubated at 37°C). In the other contaminated bottles, formula samples were analyzed after the formula mixing and after 2 hours of storage at room temperature. *Salmonella* spp and *C sakazakii* presence/absence was observed in 25 mL of product by ISO 6579-1 (11) and ISO 22964 (12), respectively, to evaluate their inactivation. The time/temperature profile in all the replicates was monitored by datalogger Thermo Button (Astori Tecnica s.n.c., Brescia, Italy).

### Data Analyses

Bacterial counts were converted to log CFU/mL. The individual means and standard deviations were determined as the average result of 2 samples for 3 replicates. When the pathogens levels were below to the detection limit (1 log CFU/mL), results were reported as presence or absence in 25 mL.

## RESULTS

The initial concentrations of *Salmonella* spp. and *C. sakazakii* artificially inoculated in formula were 1.2 ± 0.1 log CFU/mL and 1.4 ± 0.2 log CFU/mL respectively. During the simulation of the procedure suggested by FAO/WHO, the formula was prepared using hot water at 70°C. Just after formula mixing (120 seconds) and after the storage time (2 hours at room temperature), pathogens levels were below the detection limit (<1 log CFU/mL) but the presence of viable cells was observed in 25 mL of product (Table 1). Only in one replicate (replicate 2), we observed the pathogens absence in 25 mL after the formula mixing, but results were positive (presence in 25 mL) after the storage time of replicate (2 hours), indicating the cells survival during the procedure of preparation.

**FIGURE 1:**
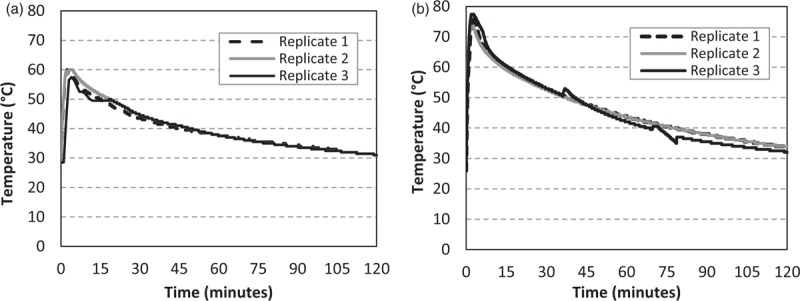
Time/temperature profiles monitored in formula reconstituted with water at 70°C (FAO/WHO) (A) and with water at 87 ± 2°C (alternative procedure) (B). Data are reported for all tests (3 replicates).

The time/temperature profiles registered during the tests are reported in Figure 1. The results show that the maximum temperature registered in 200 mL of reconstituted formula ranged between 60 and 57.5°C during the bottles mixing (Fig. 1A).

For the pathogens inactivation with a different time/temperature profile, 500 mL of boiling water was cooled at room temperature for 10 minutes and then it was added to powdered formula. Starting from water temperature at 87 ± 2°C (range of 85–89°C), the maximum temperature registered in formula was 76°C (range of 73.5–76°C) (Fig. 1B). In these conditions, no pathogens survival was observed in formula sampled after the mixing and after the storing time in all investigated replicates (Table 1).

## DISCUSSION

The contamination of low-water activity foods (eg, formula powder, egg powder, chocolate, peanut butter) with *Salmonella* is a well-known problem. It has been observed that foodborne pathogens may not grow in these food matrices, but they can survive for long periods once the food is contaminated, thus representing a significant risk even at low levels (13). Therefore, also powdered infant formula may be intrinsically contaminated with pathogens that can cause serious illness in infants. An even more challenging situation was identified for *C sakazakii* within food factories potentially available to young infants and households (14). For these reasons, *Salmonella* spp and *C sakazakii* are of major concern in the infant-food industry, because ingestion of very few (10–100 CFU/mL) *Salmonella* spp cells by young children may cause severe illness, while on the other hand *C sakazakii* may be at the origin severe infections such as meningitis, bacteriemia, and necrotizing enterocolitis, particularly in preterm, immune-compromised infants, with a death rate up to 80% and an infectious dose estimated 1000 cells (15). The recent Salmonellosis outbreak in France, Spain, and Greece due to the consumption of contaminated formula powder highlights the need to improve in any case the safety of these products and to provide more information about their correct use. International guidelines, for instance, the ESPGHAN Committee on Nutrition in 2004 (8) have been developed to diminish the risks associated with the presence of both *Salmonella* spp and *C sakazakii* in powdered infant formula, while highlighting the role of good hygiene practices in the preparation of powdered formula, its safe storage, transport, and use. Reconstitution of powdered formula for infants with hot water (no less than 70°C) has been recommended by the FAO/WHO (10) to reduce the biological risk for *Salmonella* spp. and *C sakazakii*. This procedure may, however, be considered not safe enough to guarantee the product safety because *Salmonella* spp are reported to have an increased heat tolerance at temperatures above 70°C in foods at low water activity achieved by drying (16) and it may be difficult to replicate the procedure suggested by FAO/WHO in a domestic setting.

In our study, monitoring in real time the temperature profile during formula preparation shows that even if starting from water at 70°C a rapid cooling may occur, providing non-lethal conditions for some preexisting pathogens in the powdered formula. Accordingly, *Salmonella* spp and *C sakazakii* may survive in reconstituted formula and, as a consequence, cause severe illness in infants. Just using hot water (>85°C in our study) the formula reached temperatures lethal to pathogens to reduce the associated biological risks. These results are consistent with the progressive risk reductions described in the passage from water at 50°C to reconstitution at 70°C (9). Accordingly, higher temperatures are needed to reduce the biological risk within the “real-world” associated conditions in which these products are prepared and used. Indeed, among parents of formula-fed infants <12 weeks old, only 22% were reported using water heated at ≥70°C to dilute powdered formula (17).

In conclusion, powdered infant formula could not be sterile. Industry and regulators play the primary critical role in minimizing the risk of illness from consumption of reconstituted powdered formula and ensuring that the product is safe (18). More information are needed to improve the safety of these products to propose a procedure able to guarantee the safety of the product by inactivating pathogenic bacteria and be easily feasible in actual domestic conditions. In maternity units where premature infants are looked after, sterile liquid formulae appear to be a suitable alternative to powder formulae to reduce the risk of formula-borne infections (8). Once a general agreement is reached on the optimal temperature of reconstitution, the effects on the nutrient bio-availability should be carefully evaluated.

## Figures and Tables

**TABLE 1:** Microbiological results about the *Salmonella* spp and *C sakazakii* survival in powdered formula using different procedures.

Pathogen	Inoculum level (log CFU/mL)	After formula mixing		After Storage time	
		FAO/WHO^*^	Alternative procedure	FAO/WHO	Alternative procedure
*Salmonella* spp.	1.2 ± 0.1	+ − +	− − −	+ + +	− − −
*C. sakazakii*	1.4 ± 0.2	+ − +	− − −	+ + +	− − −

When the pathogen levels were below to the detection limit (1 log CFU/mL) the presence/absence in 25 mL of formula was determined by ISO 6579-1 (11) and ISO 22964 (12).

^*^Data are reported as presence (+) or absence (−) of the pathogens in 25 mL of formula (3 replicates).
